# Supplementary material for: A Novel Sesterterpenoid, Petrosaspongin and γ-Lactone Sesterterpenoids with Leishmanicidal Activity from Okinawan Marine Invertebrates
Source: Mar Drugs. 2024 Dec 30;23(1):16. doi: 10.3390/md23010016 (PMC11766837; doi:10.3390/md23010016)
Supplement: Supplementary file 1 [file marinedrugs-23-00016-s001.zip › marinedrugs-3393391-supplementary.pdf]

Supplementary Material

A Novel Sesterterpenoid, Petrosaspongin and γ-Lactone Sesterterpenoids with Leishmanicidal Activity from Okinawan Marine Invertebrates

Takahiro Jomori[1], Nanami Higa[1], Shogo Hokama[1], Trianda Ayuning Tyas[1], Natsuki Matsuura[2], Yudai Ueda[2], Ryo Kimura[2], Nicole Joy de Voogd[3,4], Yasuhiro Hayashi[5], Mina Yasumoto-Hirose[6], Junichi Tanaka[1], and Kanami Mori-Yasumoto [2*]*

[1] Department of Chemistry, Biology and Marine Science, Faculty of Science, University of the Ryukyus, Nishihara, Okinawa 903-0213, Japan; tjomori7@sci.u-ryukyu.ac.jp
[2] Faculty of Pharmaceutical Sciences, Tokyo University of Science, Noda, Chiba 278-8510, Japan; yasumoto@rs.tus.ac.jp
[3] Naturalis Biodiversity Center, 2300 RA, Leiden, The Netherland; nicole.devoogd@naturalis.nl
[4] Institute of Environmental Sciences (CML), Leiden University, Leiden, The Netherlands
[5] Faculty of Agriculture, University of Miyazaki, 1-1 Gakuen-kibanadai-nishi, Miyazaki, Miyazaki 889-2192, Japan; hayashi_yasuhiro@cc.miyazaki-u.ac.jp
[6] Tropical Technology Plus, Uruma, Okinawa 904-2234, Japan; myhiro@ttc.co.jp

*Correspondence: TJ: tjomori7@sci.u-ryukyu.ac.jp; Tel.: +81-98-895-8560,
KMY: yasumoto@rs.tus.ac.jp; Tel.: +81-04-7121-4134

List of Contents

no.	Content	Page
1	Figure S1. ^{1}H NMR spectrum of petrosaspongin (**1**) in $CDCl_3$ (500 MHz)	2
2	Figure S2. ^{13}C NMR spectrum of petrosaspongin (**1**) in $CDCl_3$ (125 MHz)	2
3	Figure S3. HSQC spectrum of petrosaspongin (**1**) in $CDCl_3$ (500 MHz)	3
4	Figure S4. HMBC spectrum of petrosaspongin (**1**) in $CDCl_3$ (500 MHz)	3
5	Figure S5. ^{1}H-^{1}H COSY spectrum of petrosaspongin (**1**) in $CDCl_3$ (500 MHz)	4
6	Figure S6. NOESY spectrum of petrosaspongin (**1**) in $CDCl_3$ (500 MHz)	4
7	Figure S7. HR-ESI-MS data of petrosaspongin (**1**)	5
8	Figure S8. FTIR spectrum of petrosaspongin (**1**)	5
9	Figure S9. UV spectrum of petrosaspongin (**1**)	6
10	Figure S10. Docking simulation of γ-lactone sesterterpenoids with Ld-topI	7

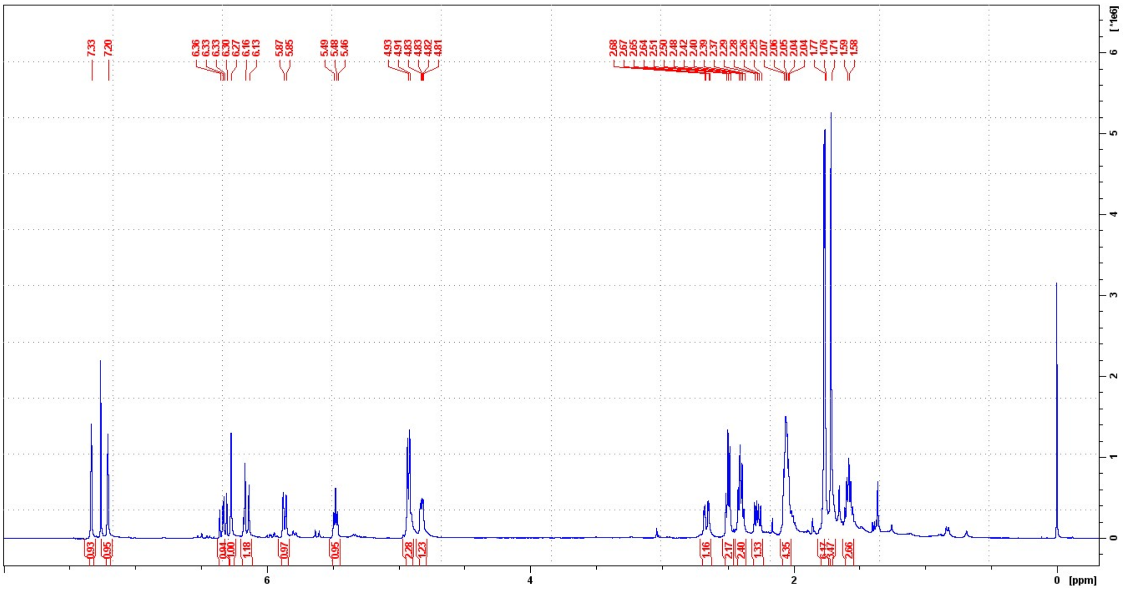

Figure S1. ^{1}H NMR spectrum of petrosaspongin (**1**) in $CDCl_3$ (500 MHz)

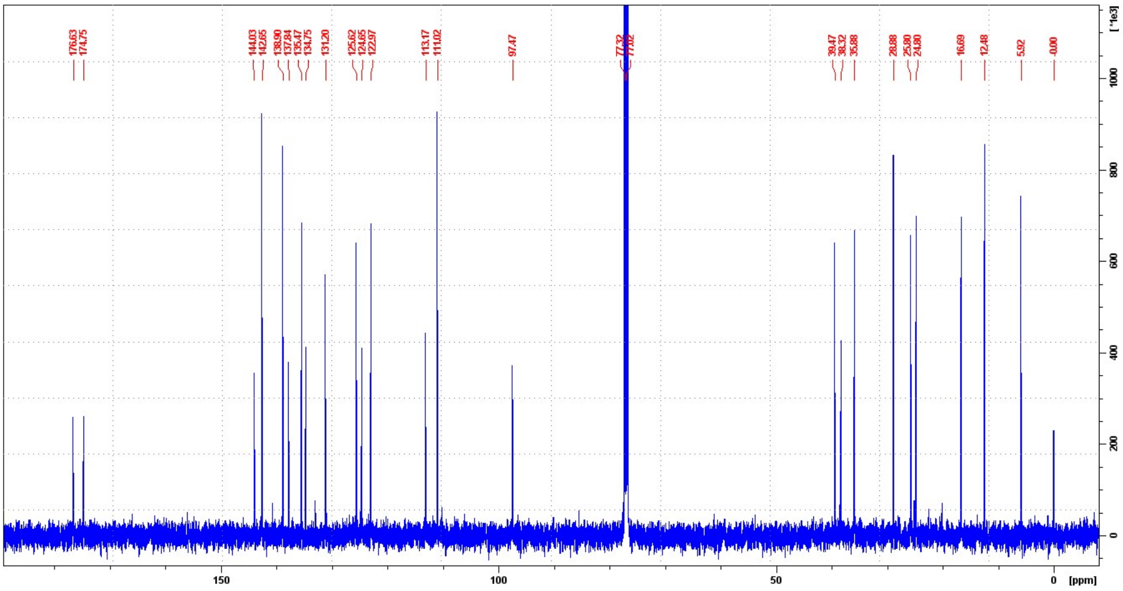

Figure S2. ^{13}C NMR spectrum of petrosaspongin (**1**) in $CDCl_3$ (125 MHz)

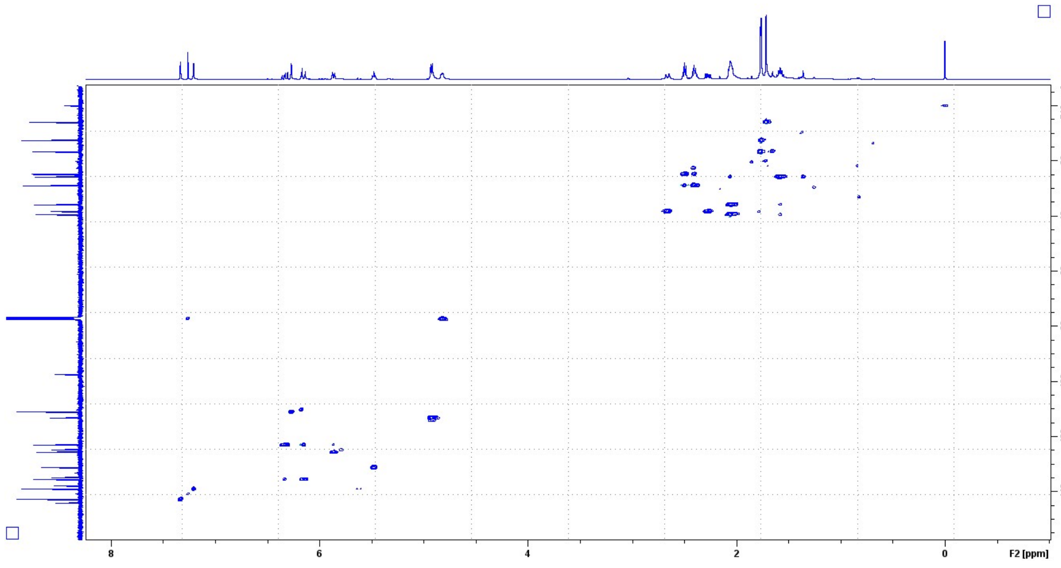

Figure S3. HSQC spectrum of petrosaspongin (**1**) in $CDCl_3$ (500 MHz)

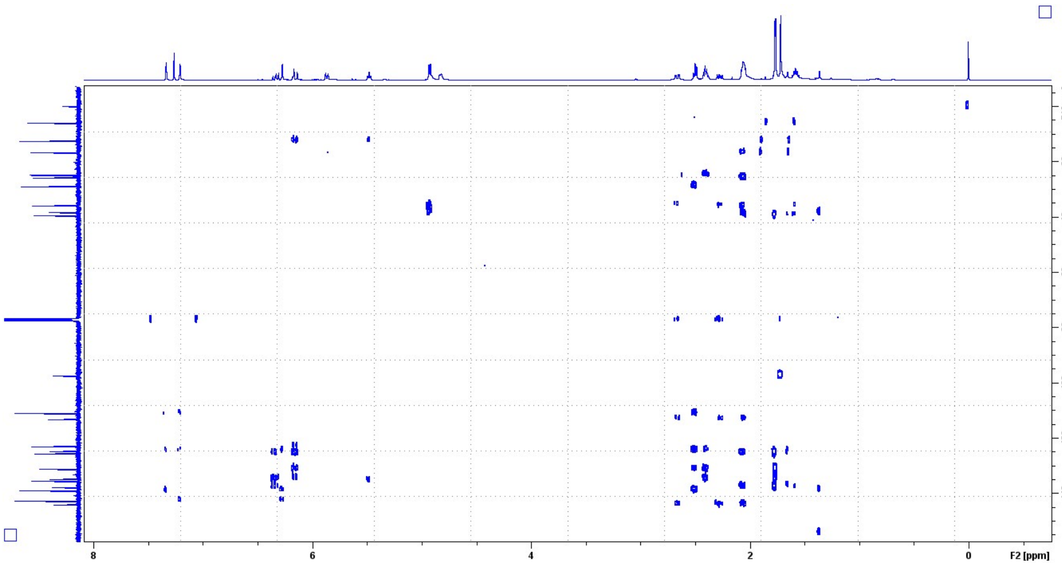

Figure S4. HMBC spectrum of petrosaspongin (**1**) in $CDCl_3$ (500 MHz)

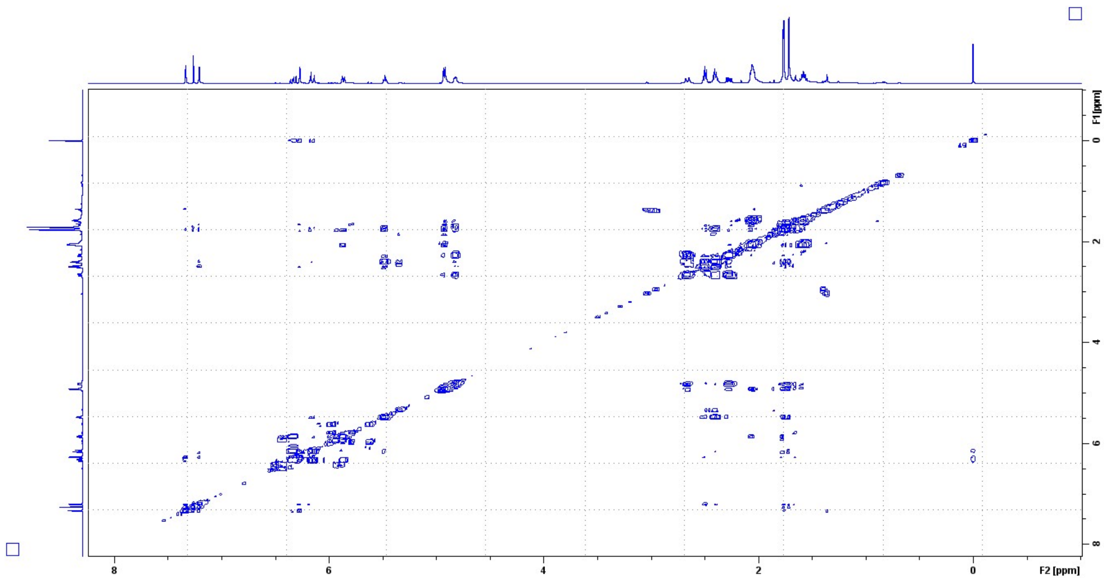

Figure S5. ^{1}H-^{1}H COSY spectrum of petrosaspongin (**1**) in $CDCl_3$ (500 MHz)

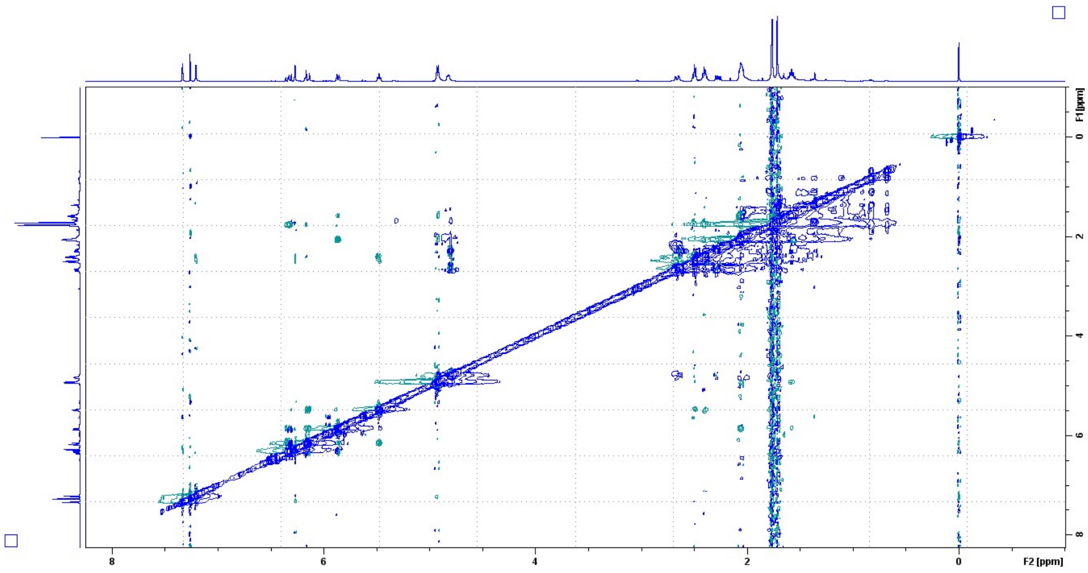

Figure S6. NOESY spectrum of petrosaspongin (**1**) in $CDCl_3$ (500 MHz)

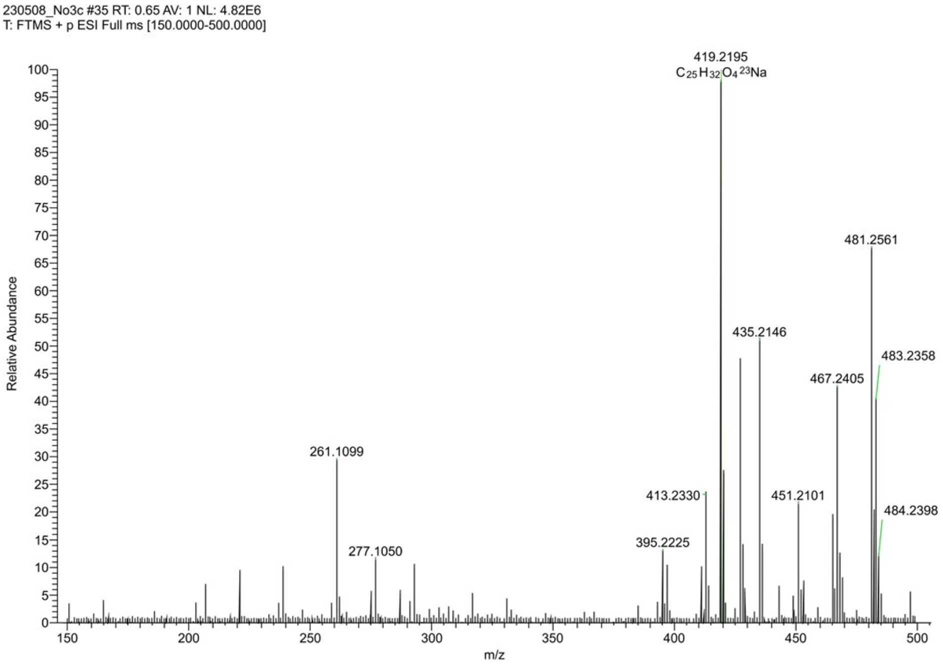

Figure S7. HRESIMS data of petrosaspongin (**1**)

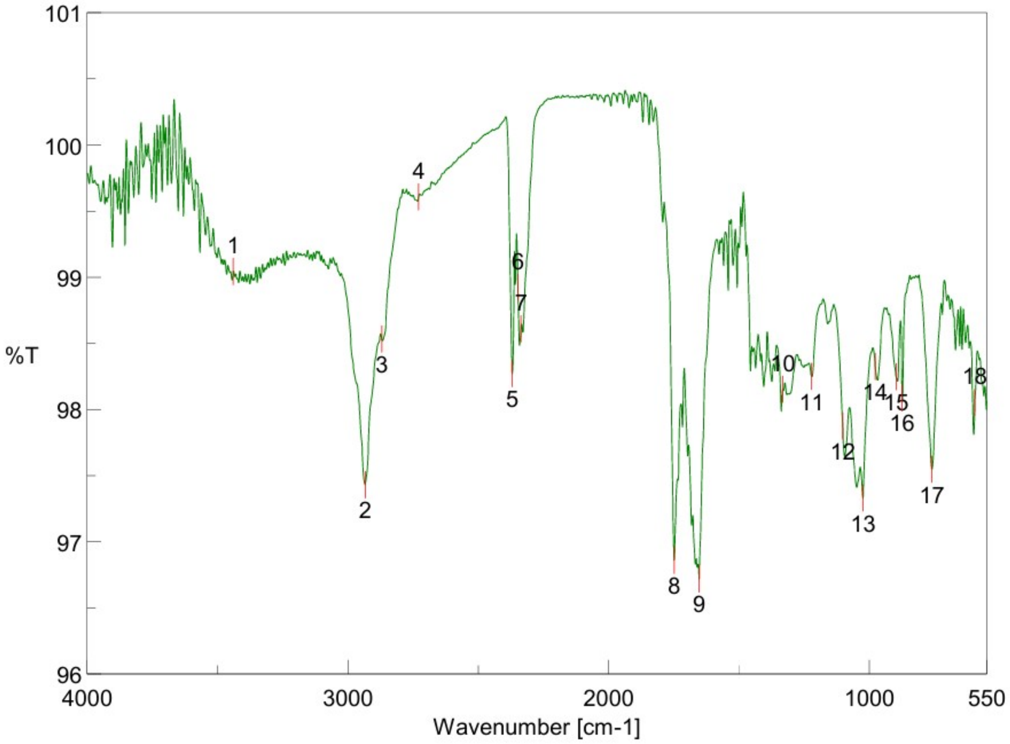

Figure S8. FTIR spectrum of petrosaspongin (**1**)

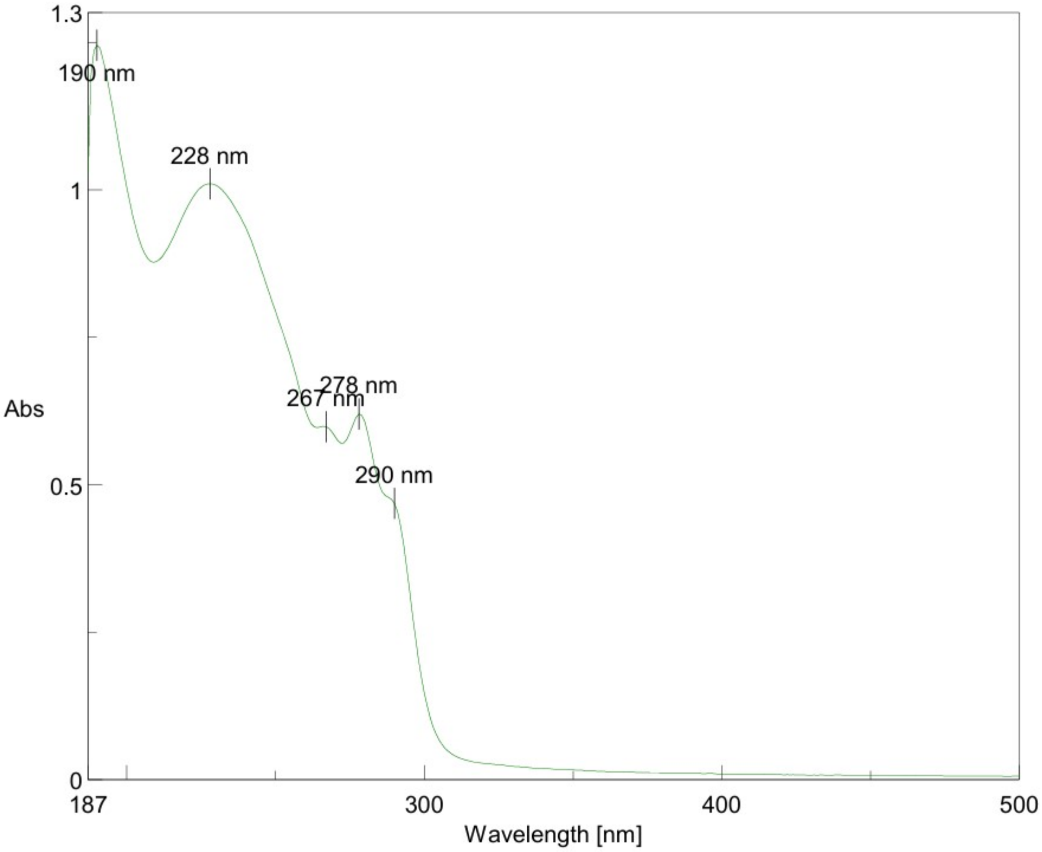

Figure S9. UV spectrum of petrosaspongin (**1**)

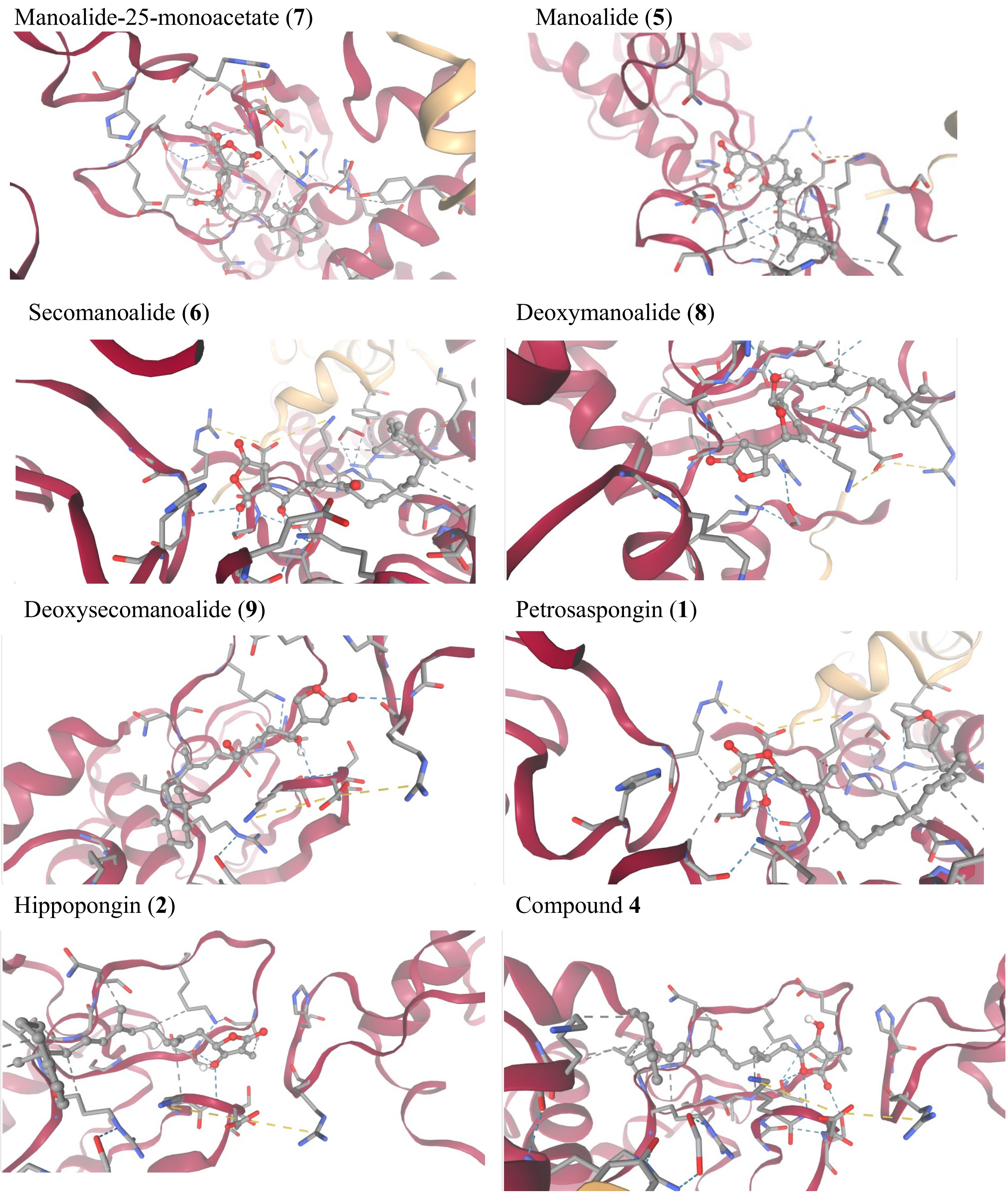

Figure S10. Docking simulation of γ-lactone sesterterpenoids with DNA topoisomerase I of *Leishmania donovani* (Ld-topI: PDB code: 2B9S). Hydrogen bonds, lipophilic interactions, and ionic interactions are represented by blue, grey, and yellow dashed lines, respectively.
